# Case report: Emergence of dolutegravir resistance in a patient on second-line antiretroviral therapy

**DOI:** 10.4102/sajhivmed.v21i1.1062

**Published:** 2020-07-02

**Authors:** Kairoonisha Mahomed, Carole L. Wallis, Liezl Dunn, Shavani Maharaj, Gary Maartens, Graeme Meintjes

**Affiliations:** 1 Private practice, Johannesburg, South Africa; 2 Department of Molecular Pathology, BARC-SA and Lancet Laboratories, Johannesburg, South Africa; 3 Aid for AIDS Management (Pty) Ltd, Cape Town, South Africa; 4 Department of Medicine, Faculty of Health Sciences, University of Cape Town, Cape Town, South Africa

**Keywords:** HIV drug resistance, antiretroviral therapy, regimens, dolutegravir, rifampicin

## Abstract

**Introduction:**

The integrase strand transfer inhibitor dolutegravir (DTG) has a high genetic barrier to resistance. Only rare cases of resistance to DTG have been reported when it is used as a component of antiretroviral therapy regimens in treatment-experienced patients unless there was prior use of a first-generation integrase inhibitor.

**Patient presentation:**

A 38-year-old woman diagnosed with tuberculosis was switched to a second-line antiretroviral regimen of zidovudine, lamivudine and dolutegravir 50 mg 12-hourly together with rifampicin-based TB treatment. Based on treatment history and a previous resistance test there was resistance to lamivudine but full susceptibility to zidovudine. The patient did not suppress her viral load on this regimen and later admitted to only taking dolutegravir 50 mg in the morning because of insomnia.

**Management and outcome:**

A second resistance test was performed which showed intermediate level of resistance to dolutegravir. Her regimen was changed to tenofovir, emtricitabine and ritonavir-boosted atazanavir with rifabutin replacing rifampicin for the remainder of her TB treatment. She achieved viral suppression on this regimen.

**Conclusion:**

To our knowledge this is the first case report from South Africa of emergent dolutegravir resistance in a treatment-experienced, integrase inhibitor-naïve patient. Factors that may have contributed to resistance emergence in this patient were that there was only one fully active nucleoside reverse transcriptase inhibitor in the regimen and lower exposure to dolutegravir because of the reduced dosing frequency while on rifampicin.

## Case presentation

A 38-year-old woman started antiretroviral therapy (ART) in 2007 with zidovudine (AZT), lamivudine (3TC) and efavirenz. Her baseline human immunodeficiency virus (HIV) viral load and cluster of differentiation 4 (CD4) cell count results were not available. In September 2009, she experienced virological failure (this HIV viral load result is not available), and a genotypic antiretroviral resistance test showed a thymidine analogue mutation (TAM, K219KE [i.e. mixed population of mutant and wild type at that codon]), M184M/V and three non-nucleoside reverse transcriptase inhibitor (NNRTI) mutations (A98G, E138A and K238T) (see Table 1).

**TABLE 1:** CD4 count and HIV viral load results available.

Date	CD4 count (cells/mm^3^)	HIV viral load (copies/mL)	ART regimen	Genotype mutations
2007	-	-	AZT, 3TC and EFV	-
04 September 2009	-	-	AZT, 3TC and EFV	NRTI resistance mutations: **M184MV** and **K219KE** NNRTI resistance mutations: **A98G, E138A** and **K238T** Other mutations: None PI major resistance mutations: None PI accessory resistance mutations: None Other mutations: M36MI, L63LAPV, T74TS, V77VI, L89LM and I93L
24 December 2009	912	24	TDF/FTC and ATV/r	-
17 June 2010	667	27	TDF/FTC and ATV/r	-
18 February 2011	1072	519	TDF/FTC and ATV/r	-
30 January 2012	850	225	TDF/FTC and ATV/r	-
19 March 2014	286	881 481	TDF/FTC and ATV/r	-
			ABC/3TC and LPV/r	-
8 June 2017	667	345 406	ABC/3TC and ATV/r	-
21 November 2017	966	2800	AZT, 3TC and DTG (50 mg bd)	-
05 February 2018	-	-	AZT, 3TC and DTG (50 mg bd)	NRTI resistance mutations: **M184V** NNRTI resistance mutations: **A98G, E138A** Other mutations: None PI major resistance mutations: None PI accessory resistance mutations: None Other mutations: E35D, L63V, T74S, V77I, L89M and I93L IN major resistance mutations: **T66TI, G118R** and **E138EK** IN accessory resistance mutations: None Other mutations: None
27 February 2018	-	10 700	AZT, 3TC and DTG (50 mg bd)	-
20 June 2018	1008	230	TDF/FTC and ATV/r	-
07 January 2019	1378	< 20	TDF/FTC and ATV/r	-
28 June 2019	1290	< 20	TDF/FTC and ATV/r	-

ART, antiretroviral therapy; CD4, cluster of differentiation 4; NRTI, nucleos(t)ide reverse transcriptase inhibitor; NNRTI, non-nucleoside reverse transcriptase inhibitor; HIV, human immunodeficiency virus; AZT, zidovudine; 3TC, lamivudine; EFV, efavirenz; TDF/FTC, tenofovir/emtricitabine; ATV/r, atazanavir/ritonavir; ABC/3TC, abacavir/lamivudine; LPV/r, lopinavir/ritonavir; DTG, dolutegravir; PI, protease inhibitor; IN, integrase inhibitor.

Based on this genotype, her regimen was changed to tenofovir (TDF), emtricitabine (FTC) and ritonavir-boosted atazanavir. She then transitioned from HIV care in the private sector to the public sector where she was changed to abacavir (ABC), 3TC and ritonavir-boosted lopinavir. When she returned to private sector care in May 2017, she reported severe diarrhoea, resulting in poor adherence to ART. Her medication was switched to ABC, 3TC and ritonavir-boosted atazanavir and the diarrhoea settled.

In June 2017, she was diagnosed with tuberculosis (TB) and was started on a rifampicin-based TB treatment. Her HIV viral load at this time was 345 406 copies/mL. As she already had significant diarrhoea on standard dose ritonavir-boosted lopinavir, it was felt that double-dose ritonavir-boosted lopinavir during TB treatment would not be tolerated. Given that AZT was still fully susceptible at the time of first-line failure, it was thought that AZT would still be susceptible and therefore providing one active nucleoside reverse transcriptase inhibitor (NRTI) to accompany dolutegravir (DTG). The patient was therefore switched to AZT, 3TC and DTG 50 mg 12-hourly. The patient informed her doctor in July 2017 that she was experiencing insomnia on this regimen. She was reassured and advised to continue until she had completed TB treatment.

There were regular pharmacy claims for treatment from June 2017 until November 2017 when her viral load was 2800 copies/mL. A resistance test was performed in February 2018, and the following mutations were observed: three integrase mutations (T66TI, G118R and E138EK) resulting in high-level resistance to raltegravir and elvitegravir and intermediate resistance to DTG (based on the Stanford score), M184V and two NNRTI mutations (A98G and E138A). A sample was also collected for phenotypic testing at monogram and the results showed that there was a 21-fold reduction in DTG susceptibility. Furthermore, all the other available integrase inhibitors had reduced susceptibility (3.92; 32- and 24-fold reduction to bictegravir, elvitegravir and raltegravir, respectively).

She then admitted to only taking DTG 50 mg in the morning because she had experienced insomnia with twice daily dosing, which resolved if she skipped the evening dose. Based on the resistance test result, her ART regimen was changed to TDF, FTC and ritonavir-boosted atazanavir in February 2018 with rifabutin replacing rifampicin for the remaining months of TB treatment. An HIV viral load performed 3 months after the change was 230 copies/mL, and subsequent viral loads have been below 20 copies/mL for about 12 months.

## Discussion

The integrase strand transfer inhibitor DTG has a high genetic barrier to resistance. In clinical trials evaluating DTG as a component of triple drug therapy in ART-naïve patients, no emergence of resistance to DTG has been reported. Only rare cases of emergence of resistance to DTG have been reported when it has been used as a component of ART regimens in treatment-experienced patients, unless there has been prior use of the first-generation integrase inhibitors, raltegravir and elvitegravir.^1^

In the case reported here of a treatment-experienced patient, DTG resistance was detected after 8 months on a regimen of AZT, 3TC and DTG. Based on the patient’s treatment history and resistance test results, this patient’s virus (at the time of starting this regimen) had resistance to 3TC (through the M184V mutation, which also re-sensitises the virus to AZT in the presence of TAMs), but was fully susceptible to AZT. The Stanford score for AZT on the 2009 resistance test was 0 (the one TAM that was present, K219E, results in potential low-level resistance to AZT, but is counteracted by the re-sensitising effect of M184V) and between 2009 and 2017, the patient received only TDF/FTC and ABC/3TC as NRTIs. These NRTI combinations typically do not select for TAMs, the mutations that may compromise AZT. In addition, there were no TAMs found on the 2018 resistance test.

Based on the Dolutegravir versus ritonavir-boosted lopinavir both with dual nucleoside reverse transcriptase inhibitor therapy in adults with HIV-1 infection in whom first-line therapy has failed (DAWNING) trial results, it would be predicted that her regimen of DTG with two NRTIs, one of which (AZT) was fully active, should be effective. The DAWNING trial evaluated DTG with NRTIs in second-line ART and demonstrated that when there was at least one fully active NRTI accompanying DTG, then this regimen was superior to ritonavir-boosted lopinavir combined with NRTIs: at week 48, 84% in the DTG arm achieved viral suppression compared with 70% in the lopinavir arm.^2^ Whilst no protease inhibitor mutations were detected in patients in the ritonavir-boosted lopinavir arm, 2/283 patients in the per-protocol analysis and in the DTG arm developed integrase resistance mutations (11 patients in the DTG arm who met virologic withdrawal criteria had a resistance test performed). One patient developed H51HY, G118R, E138EK and R263K mutations and the other patient developed the G118R mutation. Like these two cases in DAWNING, the patient reported here developed DTG resistance on second line despite there being a fully active NRTI in the regimen.

Another factor that may have contributed to the development of DTG resistance in this patient was the drug interaction with rifampicin. Rifampicin is a potent inducer of UGT1A1 and CYP3A4, the enzymes that metabolise DTG and thereby reduces concentrations of DTG (the trough DTG concentration is reduced by 85%).^3^ Studies in healthy volunteers and patients with HIV and TB have shown that this reduction can be compensated by increasing the frequency of DTG dosing from 50 mg daily to 50 mg 12-hourly in patients on rifampicin, which results in trough concentrations similar to patients taking DTG 50 mg daily without rifampicin, and resulted in adequate virological responses in patients with HIV and TB in the INSPIRING trial.^4,5^ Our patient decreased the DTG dose to 50 mg daily whilst on rifampicin because of insomnia, which would have resulted in considerably lower exposure to DTG and may have contributed to the emergence of resistance. A case has been reported in which DTG resistance emerged in a patient on DTG-containing first-line regimen who was taking rifampicin for treatment of a staphylococcal infection. The DTG was increased to 50 mg twice daily, but despite this, the patient had unexpectedly lower DTG concentrations.^6^

It is important to note that the need for this dose adjustment of DTG with rifampicin is currently being investigated. Because DTG was shown to have antiviral efficacy at doses as low as 10 mg daily in phase 2 trials,^7,8^ it is possible that a dose increase is not required in patients on first-line DTG-based ART and rifampicin. This is supported by a recent pharmacokinetic study in healthy volunteers that showed that in patients taking DTG 50 mg once daily with rifampicin, all had DTG trough concentrations above the protein-adjusted IC_90_.^3^ A recent observational study from Botswana reported similar virologic outcomes in patients on rifampicin taking DTG 50 mg 12-hourly versus 50 mg daily.^9^ The RADIANT-TB trial is being conducted in Cape Town and is enrolling patients on TB treatment and starting first-line ART who are being randomised to TDF/FTC/DTG 50 mg 12-hourly versus TDF/FTC/DTG 50 mg daily (https://clinicaltrials.gov/ct2/show/NCT03851588). Until data from this trial are available, we recommend DTG be dosed at 50 mg 12-hourly in all patients on rifampicin.

Whilst very few cases of DTG resistance have been reported in patients taking DTG as part of a triple-drug first-line ART regimen in routine clinical practice, one feature of the cases reported has been a high baseline viral load.^6,10,11^ This may also have been a contributing factor in the patient described in this case report.

In conclusion, although there is a very low risk of DTG resistance when used as part of the first-line combination therapy, DTG resistance is not uncommon after monotherapy and when used in patients previously exposed to raltegravir or elvitegravir therapy. However, apart from the DAWNING and Dolutegravir versus raltegravir in antiretroviral-experienced, integrase-inhibitor-naive adults with HIV (SAILING) trials,^2,12^ there is limited data on the risk of DTG resistance in treatment-experienced, integrase inhibitor-naïve patients, especially in routine clinical practice, with concomitant usage of rifampicin and variable adherence and when used long term. To our knowledge, this is the first case report from South Africa of DTG resistance emerging in a patient who was integrase inhibitor-naïve when starting DTG. Factors that may have contributed to resistance emergence in this patient were that there was only one fully active NRTI in the regimen in a treatment-experienced patient (based on population-based genotyping) and the lowered exposure to DTG because the patient reduced the DTG dosing frequency from 50 mg 12-hourly to 50 mg daily against her doctor’s advice whilst on rifampicin because of insomnia.

## References

[1] Rhee SY, Grant PM, Tzou PL, et al A systematic review of the genetic mechanisms of dolutegravir resistance. J Antimicrob Chemother. 2019;74(11):3135–3149. 10.1093/jac/dkz256 31280314 PMC6798839

[2] Aboud M, Kaplan R, Lombaard J, et al Dolutegravir versus ritonavir-boosted lopinavir both with dual nucleoside reverse transcriptase inhibitor therapy in adults with HIV-1 infection in whom first-line therapy has failed (DAWNING): An open-label, non-inferiority, phase 3b trial. Lancet Infect Dis. 2019;19(3):253–264. 10.1016/S1473-3099(19)30036-2 30732940

[3] Wang X, Cerrone M, Ferretti F, et al Pharmacokinetics of dolutegravir 100 mg once daily with rifampicin. Int J Antimicrob Agents. 2019;54(2):202–206. 10.1016/j.ijantimicag.2019.04.009 31002950

[4] Dooley KE, Sayre P, Borland J, et al Safety, tolerability, and pharmacokinetics of the HIV integrase inhibitor dolutegravir given twice daily with rifampin or once daily with rifabutin: Results of a phase 1 study among healthy subjects. J Acquir Immune Defic Syndr. 2013;62(1):21–27. 10.1097/QAI.0b013e318276cda9 23075918

[5] Dooley KE, Kaplan R, Mwelase N, et al Dolutegravir-based antiretroviral therapy for patients co-infected with tuberculosis and HIV: A multicenter, noncomparative, open-label, randomized trial. Clin Infect Dis. 2020;70(4):549–556. 30918967 10.1093/cid/ciz256

[6] Pena MJ, Chueca N, D’Avolio A, Zarzalejos JM, Garcia F Virological failure in HIV to triple therapy with dolutegravir-based firstline treatment: Rare but possible. Open Forum Infect Dis. 2018;6(1):ofy332 10.1093/ofid/ofy332 30631792 PMC6324549

[7] Min S, Sloan L, DeJesus E, et al Antiviral activity, safety, and pharmacokinetics/pharmacodynamics of dolutegravir as 10-day monotherapy in HIV-1-infected adults. AIDS. 2011;25(14):1737–1745. 10.1097/QAD.0b013e32834a1dd9 21716073

[8] Van Lunzen J, Maggiolo F, Arribas JR, et al Once daily dolutegravir (S/GSK1349572) in combination therapy in antiretroviral-naive adults with HIV: Planned interim 48 week results from SPRING-1, a dose-ranging, randomised, phase 2b trial. Lancet Infect Dis. 2012;12(2):111–118. 10.1016/S1473-3099(11)70290-0 22018760

[9] Modongo C, Wang Q, Dima M, et al Clinical and virological outcomes of TB/HIV coinfected patients treated with dolutegravir-based HIV antiretroviral regimens: Programmatic experience from Botswana. J Acquir Immune Defic Syndr. 2019;82(2):111–115. 10.1097/QAI.0000000000002126 31335593 PMC7794061

[10] Fulcher JA, Du Y, Zhang TH, Sun R, Landovitz RJ Emergence of integrase resistance mutations during initial therapy containing dolutegravir. Clin Infect Dis. 2018;67(5):791–794. 10.1093/cid/ciy228 29933437 PMC6093998

[11] Lübke N, Jensen B, Hüttig F, et al Failure of dolutegravir first-line ART with selection of virus carrying R263K and G118R. N Engl J Med. 2019;381(9):887–889. 10.1056/NEJMc1806554 31461601

[12] Cahn P, Pozniak AL, Mingrone H, et al Dolutegravir versus raltegravir in antiretroviral-experienced, integrase-inhibitor-naive adults with HIV: Week 48 results from the randomised, double-blind, non-inferiority SAILING study. Lancet. 2013;382(9893):700–708. 10.1016/S0140-6736(13)61221-0 23830355

